# Autotaxin Implication in Cancer Metastasis and Autoimunne Disorders: Functional Implication of Binding Autotaxin to the Cell Surface

**DOI:** 10.3390/cancers12010105

**Published:** 2019-12-31

**Authors:** Olivier Peyruchaud, Lou Saier, Raphaël Leblanc

**Affiliations:** 1 INSERM, Unit 1033, Université Claude Bernard Lyon 1, 69372 Lyon, France; lou.saier@inserm.fr; 2 Centre de Recherche en Cancérologie de Marseille, Institut Poli-Calmettes, INSERM, Unit 1068, University Aix/Marseille, 13009 Marseille, France; raphael.leblanc@inserm.fr

**Keywords:** autotaxin, lysophosphatidic acid, integrins, heparan sulfate, platelets, metastasis, inflammation, osteoclast, T cells

## Abstract

Autotaxin (ATX) is an exoenzyme which, due to its unique lysophospholipase D activity, is responsible for the synthesis of lysophosphatidic acid (LPA). ATX activity is responsible for the concentration of LPA in the blood. ATX expression is increased in various types of cancers, including breast cancer, where it promotes metastasis. The expression of ATX is also remarkably increased under inflammatory conditions, particularly in the osteoarticular compartment, where it controls bone erosion. Biological actions of ATX are mediated by LPA. However, the phosphate head group of LPA is highly sensitive to degradation by the action of lipid phosphate phosphatases, resulting in LPA inactivation. This suggests that for efficient action, LPA requires protection, which is potentially achieved through docking to a carrier protein. Interestingly, recent reports suggest that ATX might act as a docking molecule for LPA and also support the concept that binding of ATX to the cell surface through its interaction with adhesive molecules (integrins, heparan sulfate proteoglycans) could facilitate a rapid route of delivering active LPA to its cell surface receptors. This new mechanism offers a new vision of how ATX/LPA works in cancer metastasis and inflammatory bone diseases, paving the way for new therapeutic developments.

## 1. Introduction

**Figure 1:**
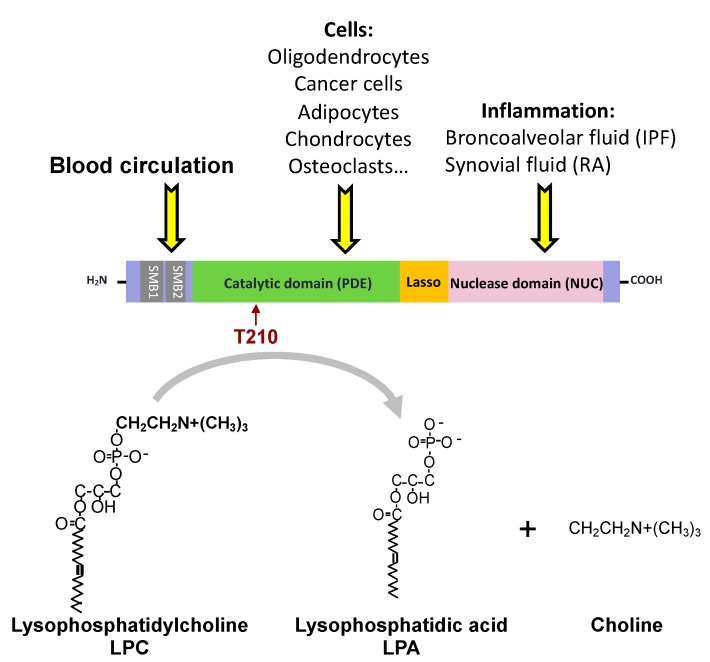
Origin, structure and enzymatic activity of autotaxin (ATX). T210 identifies the amino acid required for ATX lysophospholipase D (lysoPLD) activity. IPF, idiopathic pulmonary fibrosis; RA, rheumatoid arthritis.

The name autotaxin (ATX), proposed by Stracke and colleagues in 1992, arose during the characterization of a new potent autocrine motility-stimulating protein produced by human A2058 melanoma cells [1]. Stracke’s lab also demonstrated that ATX augments the invasive and metastatic potential of Rat Sarcoma (RAS)-transformed cells [2] before the characterization that ATX and lysophospholipase D (lysoPLD) actually correspond to the same protein [3,4]. ATX lysoPLD activity leads to the production of lysophosphatidic acid (LPA) through the degradation of a series of lysophospholipid precursors, of which lysophosphatidylcholine (LPC) is the most abundant in blood [5] (Figure 1). ATX is a multidomain protein with a somatomedin-B (SMB1,2)-like domain, a central phosphodiesterase catalytic domain (PDE), and a C-terminal inactive catalytic nuclease domain (NUC) (Figure 1). LPA exhibits growth factor-like activity due to the activation of a series of six different G protein-coupled receptors (Table 1). Expression of *ENPP2*, the ATX gene, is regulated by cytokines, growth factors and hormones (Table 2).

ATX is secreted in the extracellular space. Mechanisms responsible for ATX secretion were characterized in adipocytes showing the requirement of post-translational maturation of the N-terminal signal peptide of ATX and N-glycosylation of amino acids N53 and N410 [22]. In physiological conditions, ATX is most commonly expressed in the brain and adipose tissues. ATX and LPA are found at high levels as soluble molecules in the blood and serum and in a wide variety of pathological liquids such as synovial fluids in rheumatoid arthritis (RA) and bronchoalveolar fluid in idiopathic pulmonary fibrosis (IPF). As a consequence, ATX and LPA have been designated as key soluble mediators. However, the exact role and mechanism of action of both LPA and ATX as systemic factors in the blood, and in any other type of fluid, are still not clearly understood. Part of our misunderstanding was due to the revelation that the levels of both LPA and ATX in the bloodstream were tightly buffered, suggesting that extreme changes in concentration levels could be detrimental for the homeostasis of essential functions in the organism. In the blood, the half-lives of both LPA and ATX are remarkably short due to rapid clearance in the liver less than two minutes for ATX and around three minutes for LPA in live mice due to a high rate of degradation by cell surface lysolipid-phospholipases [23,24]. This could explain why LPA requires docking to a carrier protein in order to be protected and delivered in an active form to its receptors. In the bloodstream, LPA is largely associated with the albumin fraction [25]. It has also been shown that plasma gelsolin binds to LPA, giving it advantages for adequate presentation to its cognate receptors [8]. In this review, we will discuss recent works supporting a novel concept regarding ATX’s biological function that is dependent on its binding to the cell surface through interaction with a series of adhesion molecules. This new vision of the ATX/LPA axis could have a remarkable impact on our understanding, as well as potential therapeutic development, concerning cancer metastasis and beyond.

## 2. Autotaxin/LysoPLD Activity and Cancer Progression

The role of ATX/lysoPLD in tumorigenesis and cancer cell invasion has been well documented. Overexpression of ATX in RAS-mutated NIH3T3 murine fibroblasts increased tumor development and invasiveness compared to mock-transfected cells or cells transfected with a mutated inactive form of ATX (ATX/T210A) [2]. The Mills group has also shown that Mouse mammary tumor virus (MMTV)-ATX-tg, MMTV-LPA1-tg and MMTV-LPA3-tg animals exhibit the same phenotype by inducing development of spontaneous breast tumors. These results provide evidence supporting the contention that aberrant expression of LPA receptors, or the enzyme producing LPA, could contribute to the initiation and progression of human breast cancer [26]. Although a high expression of the ATX gene (*ENPP2*) correlates with a poor outcome in several types of cancer (such as B-cell lymphomas, renal cell carcinomas, liver or pancreatic cancers [27,28,29]), it is now well established that the tumor microenvironment is an essential source of ATX [30]. Our group notably provided evidence that circulating non-tumoral ATX is stored in the α-granules of resting platelets and is eventually released upon tumor cell-induced platelet aggregation, leading to the production of LPA [31,32]. Brindley’s studies have also suggested that inflammatory cytokines and chemokines released from breast or thyroid cancer cells induce ATX expression in tumor-associated fibroblasts and adipocytes, which in turn increases tumor progression [33,34].

ATX lysoPLD/LPA axis upregulation is not only related to tumor growth, but also contributes to other critical aspects of cancer biology including inflammation, angiogenesis, invasion and metastasis [35,36,37]. Using a human breast cancer model, we showed that ATX overexpression in ATX-null MDA-B02 human breast cancer cells increases their invasion potential in vitro and bone metastasis formation in vivo [38]. However, we observed that although inhibiting endogenous expression of ATX in mouse mammary 4T1 cells following transfection with small hairpin interference RNAs (siRNAs) affected cell invasion in vitro and reduced spontaneous metastasis dissemination to the lungs and bones in vivo, downregulating ATX does not affect the growth of primary tumors [38]. These observations suggest that in this model the autocrine function of ATX could prevail mainly on cell motility and invasion over tumor cell proliferation in vivo. The ATX/LPA axis also appears to play an important role in therapy resistance. *ENPP2* has indeed been identified as a candidate gene causing drug resistance in the long-term treatment of ovarian cancer, and stable ectopic expression of ATX in OVCAR-3 ovarian cancer cells delays apoptosis induced by carboplatin [39]. Several studies even propose that the levels of ATX in tumors and/or serum could constitute a biomarker of cancer aggressiveness. The serum level of ATX of patients with follicular lymphoma correlates with tumor burden and a poor clinical outcome [27]. It has been recently reported that ATX gene expression is significantly higher in neoplastic endometrium compared with normal tissue, especially in type I endometrial cancer [40]. Shao and colleagues have recently examined the alteration of serum ATX in 112 patients with breast cancer and 50 healthy volunteers by measuring serum ATX antigen via an ELISA assay. Interestingly, increased serum ATX was associated with breast cancer nodal status, tumor–node–metastasis (TNM) stage and Ki-67 index [41]. Although *ENPP2* mRNA expression was found to be significantly downregulated in lung cancer samples, both immunohistochemistry analysis of lung tissue biopsies and serum ATX activity levels revealed that lung cancer in humans is associated with increased levels of ATX protein and its activity [42].

## 3. Pharmacological Inhibition of ATX/LysoPLD Activity in Cancer Models

Several studies are underway to assess the therapeutic potential of ATX lysoPLD inhibitors (Table 3). Since LPA inhibits the lysoPLD activity of ATX, lipid analogs have been initially used as inhibitors [43]. While osteoclast differentiation was enhanced in the presence of MDA-B02/ATX cell-conditioned media, treatment with the LPA analog VPC8a202 significantly blocked this effect in vitro [38]. Ferry and colleagues have also described a potent ATX inhibitor, a carbacyclic phosphatidic acid analog (S32826), that possesses nanomolar activity in vitro. Due to poor bioavailability, this compound failed to show activity in animals [44]. By performing hydrolysis of the amide bond present in the S32826 compound, Tigyi’s group has developed two powerful lysoPLD inhibitors (BMP-22 and BMP-30a) that significantly decrease lung metastasis of B16-F10 syngeneic mouse melanoma [45]. Gotoh and colleagues have also demonstrated that BMP-22 reduces the number of lung metastases of B16-F10 melanoma [46] and our group has shown that BMP-22 greatly reduces the number of bone metastases [32]. However, all these lipid analogs have a limited bioavailability and efficiency in vivo. Novel small non-lipid molecule inhibitors have better oral bioavailability and induce a rapid decrease in plasma levels of LPA in murine models of inflammation [47,48]. Indeed, PF-8380, a piperazinylbenzoxazolone derivative that was the first compound shown to reduce plasma LPA levels in vivo, abrogates radiation-induced Protein kinase B (AKT) activation, and decreases tumor vascularity and tumor growth [49]. Finally, Brindley’s group have shown for the first time that systemic treatment with a tetrahydrocarboline derivative and pharmacological blocker of ATX/lysoPLD (ONO-8430506) delays early growth of 4T1 primary tumors that normalize twelve days after cell injections [50]. In agreement with previous observations based on silencing ATX expression in 4T1 cells, Benesch and colleagues observed using this model that pharmacological blockade of ATX/lysoPLD with ONO-8430506 partially inhibits spontaneous lung metastasis formation [50]. More recently, another ATX/lysoPLD inhibitor, GLPG1690, succeeded in halting the progression of idiopathic pulmonary fibrosis in Phase 2a clinical trials and it is now being tested in a Phase 3 trial [51]. In the breast cancer context, this compound has also been shown to increase radiotherapy efficiency and chemotherapy in the 4T1 mouse model [52]. However, although these inhibitors are really promising, they only partially block metastatic spread and new approaches will therefore have to be considered.

## 4. ATX Binding to the Cell Surface May Have Functional Impacts on Cell Biology and Pathophysiology

**Figure 2:**
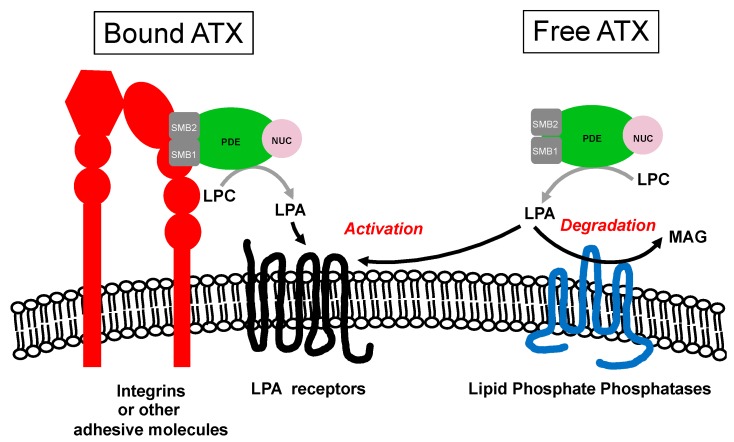
Potential mechanism for optimum activation of LPA receptors mediated by bound ATX to integrins or other cell surface adhesive molecules. This mode of action suggests that LPA generated by free ATX might be more accessible and susceptible to degradation by lipid phosphate phosphatases. LPA, lysophosphatidic acid; LPC, lysophosphatidylcholine; MAG, monoacylglycerol; SMB, somatomedin-B.

LPA is a very simple lipid subjected to strong degradation in the extracellular medium by a family of three enzymes called the lipid phosphate phosphatases (LPPs). At the plasma membrane, the catalytic site of the LPPs is oriented to the extracellular environment, enabling them to access and hydrolyze extracellular LPA [59]. Binding of ATX to the cell surface provides a mechanism for a localized production of LPA close to its receptors and away from LLPs, avoiding its degradation (Figure 2).

**Figure 3:**
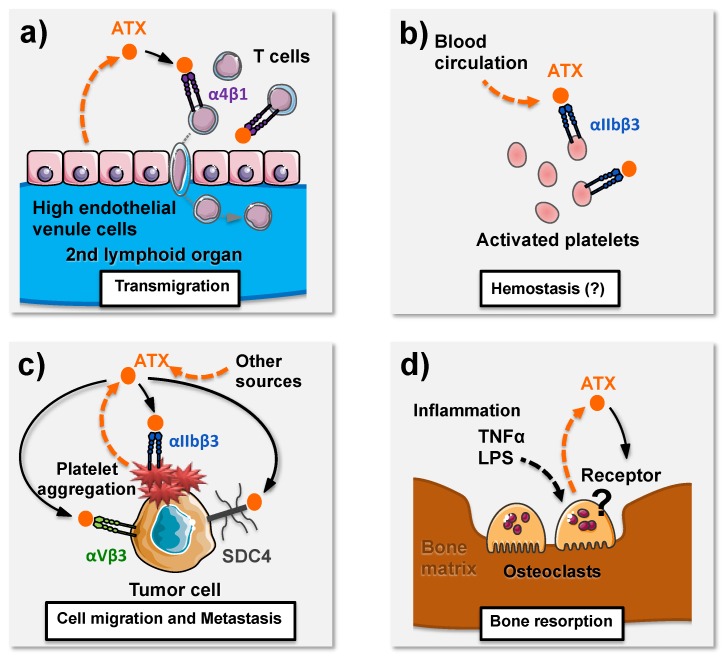
The interaction of ATX with cell surface adhesive molecules is involved in various biological processes. (**a**) ATX produced by high endothelial venule cells interacts with α4β1 integrins to promote T cell transmigration and colonization of secondary lymphoid organs. (**b**) ATX interacts with platelets though αIIbβ3 integrins. This interaction could potentially contribute to hemostasis. (**c**) ATX secreted during platelet aggregation interacts with αvβ3 integrin and syndecan 4 (SDC4) to promote cancer cell metastasis. (**d**) ATX secreted by osteoclasts under inflammatory conditions (TNFα, LPS) acts as an autocrine factor through unidentified receptors to promote bone resorption.

SMB domains present in the ATX primary sequence are small cysteine-rich domains known to mediate protein–protein interactions, an example being the vitronectin SMB domain that binds the complex between urokinase-type plasminogen activator (uPA) and its glycolipid-anchored receptor (uPAR) [60]. Thus, several teams were interested very early on in the binding capacity of ATX to the cell surface. Kanda and coworkers found that activated primary human T cells adhered to immobilized ATX through a α4β1-dependent mechanism [61]. This study also showed that T cells do not secrete ATX in contrast to endothelial cells from high endothelial venules (HEV) but binding of ATX produced by HEV cells to the T cell surface promoted the entry of the lymphocytes into secondary lymphoid organs (Figure 3a). This was likely due to the local production of LPA because intravenous injection of the enzymatic inactive form of ATX (ATX-T210A) inhibited T cell colonization by competing with the binding of endogenous active ATX to T cell surface, thereby acting as a dominant negative [39]. A study on the role of ATX/LPA in murine thrombosis showed that activated platelets are able to bind recombinant ATX through a β3 integrin-dependent mechanism [62] (Figure 3b). Binding to β3 integrin has also shown to enable the uptake and intracellular sequestration of ATX, which redistributes to the tip of migrating MDA-MB-231 breast cancer cells. In this model, ATX binding to integrins and lysoPLD activity closely cooperate to promote rapid persistent directional cell migration [63] (Figure 3c). Similar mechanisms could also be responsible for the uptake of circulating ATX by blood platelets, its storage into the α-granule compartment and eventually its release following platelet aggregation [32]. We demonstrated that this mechanism is responsible for an ATX-driven mechanism of metastasis formation in bone tissue by human breast cancer ATX-null MDA-B02 cells. We also demonstrated that the pro-tumoral activity of circulating ATX derived from platelets was mainly dependent on its interaction with tumoral αvβ3 integrins [32] (Figure 3c). In this study, we confirmed the mentioned observation of Kanda and colleagues on T cell colonization by showing that treating animals with the catalytic inactive form of ATX acts as a dominant negative, which in our case is inhibition of the formation of bone metastases at an early stage, reinforcing the importance of maintaining ATX on the cell surface for functional involvement of lysoPLD activity in pathophysiological systems [32].

Crystallographic studies of ATX have been completed, revealing a complex organization: the PDE core domain interacts with both the SMB domains on one side and the NUC domain on the other [60,64]. While SMB1 is involved in the formation of the tunnel, Hausmann and Fulkerson both observed that only the SMB2 domain is engaged in the binding of partners. It was notably shown that ATX–platelet–αIIbβ3 integrin interaction is mediated by the SMB2 domain. Although the SMB2 domain contains a canonical Arginin-Glycin-Aspartic acid (RGD) integrin-binding motif, site-directed mutagenesis studies revealed that the ATX–integrin interaction is RGD-independent [32,62,65]. Interestingly, Fulkerson and colleagues also found that ATX increases thrombin-stimulated LPA production by washed platelets and provided evidence that ATX-mediated LPA production is significantly higher in CHO cells transfected to express αIIbβ3 integrin. This observation suggested for the first time that binding of ATX to specific partners was able to modulate its lysoPLD activity. In support of this hypothesis, blocking the ATX/αIIbβ3 interaction by performing site-directed point mutations in the SMB2 domain or by using the 7E3 antibody led to a decrease in LPA production [65].

Integrins are not the only adhesive molecules involved in maintaining ATX at the cell surface. Moolenaar’s team found that ATXα, the less abundant isoform, binds to cell surface heparan sulfate (HS) proteoglycans through a unique insertion of a 52 polybasic amino acid sequence generated following alternative splicing of the *ENPP2* gene. This insertion directs ATXα to the plasma membrane, thereby ensuring a localized LPA production and signaling [66]. Our group recently identified HS proteoglycan syndecan-4 (SDC4) as a new molecule that controls ATXβ interaction with the cancer cell surface through a domain located in the SDC4 core protein (Figure 3c). We notably found that a pretreatment with anti-SDC4 antibodies and silencing of SDC4 expression totally abolished human osteosarcoma MG63 cell proliferation induced by exogenous ATXβ. Despite that, we could not demonstrate direct interaction between SDC4 and ATX. Our results strongly support the hypothesis that the physical interaction of ATXβ with adhesive molecules induces the functional changes in ATX required for LPA biological functions [67]. In addition to binding to the cell surface, Jethwa and colleagues have suggested that ATX can bind to the surface of cell-secreted exosomes [68]. Even though the binding mechanism remains unknown, mass spectrometry analysis of lipids has revealed that exosome-bound ATX is catalytically active and carries generated LPA. Once bound to a cell, through specific integrin interactions, ATX releases the LPA which in turn promotes cell migration [68]. Taken together, these results strongly support the hypothesis that physical interaction of ATX with cell surface molecules is required for LPA biological function and can be considered as a promising target for drug development.

## 5. Inflammation-Induced Bone Loss is A Pathological Process Controlled by ATX Autocrine Action on Osteoclasts

Rheumatoid arthritis is a human autoimmune disease characterized in the clinic by highly inflammatory joints that cause swelling and destruction of both cartilage and bone [69]. Molecular mechanisms responsible for bone destruction result from the production of high levels of inflammatory cytokines in the joint cavity, of which the most powerful is TNFα, which acts directly on osteoclasts, thereby stimulating their resorption activity [70]. As opposed to inflammation, bone erosions constitute a key and irreversible outcome in RA [71]. Controlling synovial inflammation by the use of anti-TNFα monoclonal antibodies is the standard treatment for patients with RA because it can arrest inflammation and the progression of bone erosions. However, RA patients in sustained clinical remission or low disease activity often continue to accrue bone erosions, suggesting that additional mechanisms might take place within the osteoarticular environment [72]. In this context, LPA and ATX could play important roles.

LPA is detected at high levels in the synovial fluid of RA patients due to the elevated expression of ATX, mediated at least in part through TNFα action on synovial fibroblasts [73,74,75]. As a systemic consequence, ATX concentration in the serum of RA patients is increased. The serum concentration of ATX is also increased in arthritic transgenic mice expressing human TNFα (*hTNF*^+/−^), mimicking the human pathogenesis. Conditional knockout of the ATX gene, *enpp2*, in mesenchymal cells in Col6a-Cre^+^ mice, mainly affecting synovial fibroblasts, attenuated the development of arthritis [75]. This suggests a paracrine action of LPA produced by ATX derived from fibroblasts in the pathogenesis of arthritis. A hypothesis emanating from this report could predict attenuation of the inflammation by ATX inhibitor treatment. However, we found that treatment with the ATX inhibitor BMP22 did not significantly affect articular inflammation in *hTNF^+/−^* mice [15]. The simplest explanation would be that the drug had been provided at a suboptimal dosing regimen but we found that BMP22-treated *hTNF^−/−^* mice displayed a significant reduction of local bone erosion. Thus, pharmacological inhibition of ATX using BMP22 causes distinctively different effects on inflammation versus bone resorption as it does not protect *hTNF*^+/−^ transgenic mice from inflammation or swelling but protects them from inflammation-induced bone loss by reducing osteoclastic bone resorption.

We previously demonstrated that LPA controls the bone resorption activity of osteoclasts by a direct activation of its cell surface receptor LPA_1_ [38,76]. Global *lpar1* knockout mice have been shown to be protected against collagen-induced arthritis (CIA) by reducing bone destruction and infiltration of pro-inflammatory T helper (Th)17 lymphocytes into the joints [73]. LPA_1_ is the most ubiquitous LPA receptor. As a consequence, global *lpar1* knockout animals exhibit multiple defects, particularly in their neural and metabolic systems [77,78,79]. For this reason, they cannot be used in RA to distinguish between the systemic influence of inflammation and the local action of ATX/LPA in bone erosion.

Through in vitro studies, we recently demonstrated that ATX is upregulated during the course of osteoclastogenesis, and as such, ATX belongs to late osteoclastic markers [15]. In addition, we demonstrated that osteoclast-derived ATX was fully active on mature osteoclasts as shown by treatment with LPC alone, which was able to rescue osteoclastic bone resorption activity induced by Macrophage colony-stimulating factor/Receptor activator nuclear κB ligand (MCSF/RANKL) in the presence of charcoal-treated serum. By crossing *enpp2* floxed mice with Ctsk-Cre mice, we generated ∆*ATX^Ctsk^* animals with specific ablation of ATX expression in mature osteoclasts. As expected, ∆*ATX^Ctsk^* mature osteoclasts were defective in mineral matrix degradation in vitro but this was potently rescued by adding exogenous ATX, suggesting that the environment can supply ATX if necessary under specific pathophysiological conditions. In this context, variable sources of ATX and/or LPA are potentially available in the bone microenvironment with the presence of fibroblasts [75], chondrocytes [80], osteoblasts [81], adipocytes [82] and endothelial cells [61]. However, we found that ∆*ATX^Ctsk^* mice were remarkably protected against bone degradation induced by two independent powerful inflammatory challenges such as lipopolysaccharide (LPS) treatment and transfer of the serum from mice expressing the transgenic T cell receptor (TCR) and the MHC class II allele Ag7 (K/BxN), while, as expected, specific inhibition of ATX expression in mature osteoclasts does not affect the onset and progression of the inflammatory process or the accumulation of ATX in the synovitis of ∆*ATX^Ctsk^* mice [15]. These results provide the first experimental demonstration that ATX controls a pathophysiological process through autocrine action. More specifically, these results demonstrate the leading role for an autocrine action of osteoclast-derived ATX over that of synovial fibroblasts or other sources of ATX in RA responsible for bone loss (Figure 3d).

Bone metastases frequently form at advanced stages in a large range of solid tumors such as breast and prostate cancers, which are the most osteophyle cancers, but bone metastases are also secondary sites for tumor cells that have escaped from primary lung, thyroid and renal cancers [83]. In this context, osteoclasts play a crucial role because they are the only cells in the organism capable of degrading the bone matrix [83]. Osteoclast activity is also responsible for bone erosions in other types of cancers as observed in multiple myeloma [84]. We have shown that treatment with the ATX inhibitor BMP-22 reduced the progression of osteolytic lesions in mice harboring pre-established bone metastases, which indicate the inhibition of osteoclast activity [32]. It is well accepted that the development of cancer shares common characteristics with inflammatory processes due to the production of inflammatory cytokines and the infiltration of immune cells. [85]. Our studies revealed that the autocrine activity of ATX on osteoclasts could be considered a common characteristic responsible for bone loss in bone metastases and inflammatory bone diseases. We have previously demonstrated that LPA controls osteoclast differentiation and bone resorption activity by acting on its receptor, LPA_1_ [76]. However, the mode of action of ATX on osteoclasts has not been fully characterized (Figure 3d).

Mature osteoclasts express multiple adhesive molecules that have been described to interact with ATX (i.e., integrins, heparan sulfate proteoglycans). One of these, the integrin αvβ3, is a marker of osteoclast differentiation [86]. In addition, mature osteoclasts are the human cells that express this integrin the most. As a consequence, the question of whether ATX/LPA action on osteoclasts relies on the link of ATX to integrin αvβ3 merits specific investigation.

## 6. Conclusions

In conclusion, all these results suggest that binding of ATX to the cell surface through its interaction with different adhesion molecules could achieve two major objectives. The first objective is to allow the production of LPA in the immediate vicinity of its membrane receptors. LPA is highly sensitive to degradation by phospholipases that are extensively represented at cell surfaces and in biological liquids. Therefore, LPA requires protection that can be provided by docking to a carrier protein. ATX associated to the cell membrane loaded with LPA might offer the fastest way to deliver active LPA to its cell surface receptors.

The second objective is to increase the production of LPA through the amplification of ATX/lysoPLD activity. This unexpected finding was confirmed from a series of recent independent studies opening new opportunities for the future design of ATX inhibitors. Targeting the ATX-bound lysoPLD instead of the ATX-soluble lysoPLD may provide more efficient therapeutic drugs. In addition, instead of targeting the enzymatic activity of ATX, the development of drugs directed against the binding domains of ATX or to ATX adhesion molecules could be considered. These new therapeutic strategies could have several advantages. They could compensate for the mode of action of current ATX inhibitors that all induce a massive decrease in LPA concentration in the bloodstream, which during long-term treatments can potentially provoke unknown side effects with a major concern for the central nervous system and the reproductive system. Collectively, the works described in this review show a fairly wide diversity in cell surface “receptor-like” interactions for ATX. Because of the large variety of cells in the organism that express the specific pattern of adhesive molecules and as a consequence specific types of “receptor-like” molecules for ATX, it is conceivable to consider the development of new blockers of ATX action that could restrict the therapies to specific sub-types of cells and tissues.

## Figures and Tables

**Table 1:** Characteristics of lysophosphatidic acid (LPA) receptors.

Receptors	G Proteins	Cellular Responses
**LPA_1_/Edg2**	G_i/o_, G_q/11_, G_12,13_	Neurite retraction [6,7], AC inhibition [8], SRE activation [6], increased [Ca^2+^]i, IP production, MAPK activation [8], stress fiber formation, BrdU incorporation [6], inhibition of apoptosis, arachidonic acid release [8].
**LPA_2_/Edg4**	G_i/o_, G_q/11_, G_12,13_	Neurite retraction [8], AC inhibition [9], SRE activation, increased [Ca^2+^]i [9], IP production [8], MAPK activation [8], stress fiber formation, BrdU incorporation [6], inhibition of apoptosis, arachidonic acid release [8].
**LPA_3_/Edg7**	G_i/o_, G_q/11_	AC inhibition [8], increased [Ca^2+^]i, IP production, MAPK activation, arachidonic acid release [8].
**LPA_4_/p2y9/GPR23**	G_q/11_, G_12/13_, G_s_, (G_i_)	AC stimulation, increased [Ca^2+^]i [10], zif268 activation, neurite retraction, cell aggregation [10], stress fiber formation [11].
**LPA_5_/GPR92/GPR93**	G_q/11_, G_12/13_	AC stimulation, increased [Ca^2+^]i, IP production, neurite retraction [7].
**LPA_6_/p2y5**	G_12/13_, (G_s_), (G_i_)	CRE activation, neurite retraction, membrane shedding [10].

AC, adenylate cyclase; BrdU, bromodeoxyuridine; CRE, cAMP response element; IP, inositol phosphate; MAPK, mitogen-activated protein kinase; SRE, serum response element; [Ca^2+^], intracellular calcium concentration.

**Table 2:** Regulation of *ENPP2* expression.

External Signals	Transcription Factors	Effects	Cell Types	References
EGF	nd	Upregulation	Thyroid cancer cells	[12]
β-FGF	nd	Upregulation	Thyroid cancer cells	[12]
IL-4	nd	Downregulation	Fibroblast-like synoviocytes	[13]
IL-1β	nd	Downregulation	Fibroblast-like synoviocytes	[13]
BMP4	nd	Upregulation	Primed pluripotent stem cells	[14]
TNFα	NF-κB	Upregulation	Osteoclasts Transformed fibroblasts	[15] [16]
LPS	NF-κB	Upregulation	Osteoclasts	[15]
WNT	β-Catenin	Upregulation	Wilms tumors	[17]
α6β4	NFAT1	Upregulation	MDA-MB-435 cells	[18]
Glucocorticoid	nd	Upregulation	MC3T3-E1 cells	[19]
Retinoic acid	nd	Upregulation	N-myc-amplified neuroblastoma cells	[20]
------	v-jun	Upregulation	Chick embryo fibroblasts	[21]

EGF, Epidermal growth factor; β-FGF, Fibroblast growth factor-beta; IL-4, Interleukin 4; IL-1β, Interleukin 1 beta; BMP4, Bone morphogenic protein 4; TNFα,Tumor necrosis factor alpha; LPS, Lipopolysaccharide; WNT, Wingless integration site; α6β4, Intergrin α6β4; NF-κB, Nuclear factor κB; NFAT1, Nuclear factor activated T cells; nd, not determined.

**Table 3:** ATX inhibitors.

Drug Names	Cancer Types	Effects	Ref.
S32826	Ovarian cancer	Primary tumor growth retardation of OCAR-3 cells at high doses	[44]
BrP-LPA	Breast cancer	Primary tumor growth inhibition of MDA-MB-231 cells	[53]
BrP-LPA	Glioma	Primary tumor growth delay of GL-261 cells in combination with radiotherapy	[54]
BMP-22	Melanoma	Inhibition of lung metastasis of B16-F10 cells	[45]
BMP-30a	Melanoma	Inhibition of lung metastasis of B16-F10 cells	[45]
S32826	nd	Decreased intraocular pressure in Dutch-Belted rabbits (glaucoma)	[55]
GWJ-A-23	nd	Attenuation of idiopathic pulmonary fibrosis induced by bleaomycine treatment	[56]
PF-8380	Glioblastoma	Ameliorates the glioblastoma GL261 cell response to radiotherapy	[49]
Gintonin	Melanoma	Inhibition of lung metastasis of B16-F10 cells	[57]
VPC8a202	Breast cancer	Inhibition of lung metastasis of 4T1 cells	[58]
ONO-8430506	Breast cancer	Inhibition of tumor growth and lung metastasis of 4T1 cells	[50]
BMP-22	Breast cancer	Inhibition of bone metastasis of MDA-BO2 cells	[32]
GLPG1609		Current Phase 3 clinical trials for idiopathic pulmonary fibrosis	[51]
GLPG1609	Breast cancer	Sensitizes primary tumors of 4T1 cells to radiotherapy and chemotherapy	[52]
